# Behavioral Clusters and Lesion Distributions in Ischemic Stroke, Based on NIHSS Similarity Network

**DOI:** 10.1007/s41666-025-00197-6

**Published:** 2025-03-26

**Authors:** Louis Fabrice Tshimanga, Andrea Zanola, Silvia Facchini, Antonio Luigi Bisogno, Lorenzo Pini, Manfredo Atzori, Maurizio Corbetta

**Affiliations:** 1 Department of Neuroscience, University of Padua, Padua, 35128 Italy; 2 Padova Neuroscience Center, University of Padua, Padua, 35128 Italy; 3 Department of Information Engineering, University of Padua, Padua, 35128 Italy; 4 Information Systems Institute, University of Applied Sciences Western Switzerland (HES-SO Valais), Sierre, 3960 Switzerland; 5 Veneto Institute of Molecular Medicine, Padua, 35128 Italy

**Keywords:** Stroke, NIHSS, Machine learning, Clustering

## Abstract

Stroke, a leading cause of mortality and disability, results in diverse dysfunctions linked to brain lesion locations. The intricate relationship between lesions and symptoms often defies linear analysis methods. Unraveling these connections can yield valuable insights to enhance patient care, optimize rehabilitation strategies, and unveil fundamental principles of healthy brain function. This study introduces a novel unsupervised framework to stratify patients into clinically coherent subgroups based on behavioral symptom profiles and identify their distinct neural correlates. NIHSS assessments are modeled as ordinal feature vectors, integrating symptom prevalence, severity, and covariance patterns into a unified measure of behavioral similarity among stroke survivors. The resulting similarity network is partitioned using Repeated Spectral Clustering, which accumulates partition evidence for stable subgroup discovery. Voxel-wise lesion analysis subsequently highlights each subgroup’s collective neuroanatomical signatures. Despite being identified in a completely unsupervised manner based solely on NIHSS scores, the emergent clusters correspond to well-documented syndromes, validating the purely data-driven symptom groupings alongside established neurological knowledge. Clusters exhibit critical voxels in group-specific anatomical locations, even when average lesion maps spatially overlap, suggesting that our method disentangles functionally distinct substrates within shared vascular territories. Our workflow represents a significant methodological advancement, providing robust, clinically relevant insights into symptom phenotyping and lesion patterns. The framework’s mathematical transparency and validation against canonical knowledge underscore its potential for generalization to multimodal biomarkers and broader biomedical research. To foster reproducibility, we provide open-source code.

## Introduction

Stroke is the second leading cause of death and the third leading cause of disability worldwide [1, 2]. It occurs when there is an interruption in oxygenation to the brain, leading to cell death. In ischemic stroke, blood clots interrupt blood flow, whereas in hemorrhagic stroke, vessels break and blood diverges from its intended path. The subsequent damage to the brain of survivors can result in acute and chronic dysfunctions across sensory, motor, cognitive, and behavioral domains. For instance, focal cortical lesions in highly specialized brain regions can affect narrow sets of functions, as in Broca’s and Wernicke’s aphasias. More often, subcortical strokes damage both gray and white matter, disrupting connections across the brain beyond the lesion location. These phenomena shed light and spark debates on the functional organization of the brain.

To characterize and quantify behavioral symptoms, clinicians have developed several tests that score performance in multiple domains, such as the Montreal Cognitive Assessment (MoCA) [3], the Oxford Cognitive Screen (OCS) [4], and the National Institutes of Health Stroke Scale (NIHSS) [5, 6]. The NIHSS is a 15-item scale that rates the severity of sensory, motor, attention, and language deficits in stroke survivors. A score of 0 signifies a healthy response and the absence of a deficit, while maximum damage values range from 2 to 4, depending on the item. As an example, the motor arm test involves evaluating the ability to hold the limb against gravity; 0 is given when there is no drift (limb held for full 10 s at 45 or 90°), while 4 is given when the patient is unable to move the limb; each limb has its respective NIHSS item. The NIHSS test can be quickly administered at admission, during the acute phase, at the neuropsychological evaluation, and later on during recovery. Many studies have focused on the sum of all item scores, while analyses of the separate scores have often characterized only inter-item correlations at the population level.

Such analyses have repeatedly identified a low-dimensional structure of the behavioral deficits’ covariance, with 2 up to 5 components explaining the larger share of variance  [7–11]. These factors of variability separately correlate with lateral motor impairments, language, memory, and attention. Regularized regression models, statistical tests, and machine learning techniques have been developed to relate behavioral scores to brain scans.

Despite several studies have analyzed the relationship between lesion locations and behavioral symptoms, some delimiting aspects should be highlighted. First, many studies rely on implicit mathematical assumptions that might distort or discard information. In particular, although the use of rank correlations and tests is settled [12, 13], ordinal scores as found in NIHSS are usually treated as counts or continuous intervals [14, 15]. Alternatively, ordinal scales are reduced to dichotomous variables, e.g. $\{0,1\}$, to denote absence or presence of deficits [16, 17], or the cumulative sum of scores is thresholded to separate into classes of milder impairment and classes of worse prognosis [15, 18]. Second, the relationships among item scores [7, 8] and with lesion volumes and locations are usually modeled with linear methods, both for ordinal [19] and continuous scores [20]. Third, the scope is usually to study global, population-level associations between single deficits, rather than conditional associations and multivariate distributions of symptoms, i.e., syndromes. The former perspective, while insightful especially when developing clinical tests, is not concerned with individual-level phenotypization, nor sufficient for the prediction of groups of deficits. In this regard, it is also notable that predicting deficits from lesions aligns with the aetiological process, but reverses the chronological order of assessment and availability typical of the clinical setting.

This study introduces several innovative approaches; in detail, a data-driven process collects profiles with similar symptoms into distinct clusters, each cluster presenting a characteristic syndrome; subsequently, these syndromes are associated with specific lesion distributions. Moreover it complements previous perspectives by addressing the three issues highlighted above. First, a network model of pairwise similarities among subjects is presented and clustered, based on a conservative distance measure that respects the ordinal nature of data, better suited than traditional alternatives such as Euclidean or Manhattan/Hamming distances between score vectors. Second, cluster-specific lesion maps arise naturally from clustering analysis, by averaging lesion masks from patients in each cluster, rather than modeling correlations between latent variables derived from dimensionality reduction of behavioral and image variables. Third, a statistical evaluation refines lesion mapping, identifying lesions that distinguish behaviorally different groups. This approach assists in understanding etiology and allows to align of the complete profile of a single patient to a more general phenotype or syndrome, rather than associating a single symptom across the whole population to other single symptoms.

As mentioned in Yang et. al [21], clustering is one of the most useful methods for analyzing patient similarities for precision medicine. It groups patients into clinically meaningful subsets, which can be used for a variety of tasks, such as personalized therapies and policy making. Kim et al. [22] stress that finding similar clusters among stroke patients can be helpful from a medical perspective, as it may lead to the discovery of new patterns and more effective ways to manage stroke. Overall, clustering is an effective method for relating many patients to each other and to a few behavioral syndromes, and for relating behavioral syndromes to specific lesion locations.

### Related Works

Statistical analyses have traditionally focused on correlations of variables, covariance of variables with imaging data, and prediction of population outcomes. Examples of imaging and behavioral studies can be traced to Voxel-based Lesion-Symptom Mapping (VLSM) [23] in its univariate and multivariate forms, and to the Partial Least Squares (PLS) family of applications [20]. VLSM assigns *t*-statistics, *p*-values, and correlation scores to voxels in brain regions linked with behavioral deficits, while PLS reduces and correlates dimensions of brain and behavioral data. VLSM requires careful evaluation of its statistical assumptions and corrections [24], and focuses on associating voxels to one single symptom at a time, practically assuming independence of deficits and lesion-deficits associations. For this reason, the statistical evaluation is repeated for each symptom of interest, or a combination, according to researchers’ hypotheses and biases. PLS and Canonical Correlation Analysis approaches address associations both on the spatial and behavioral sides, but typically avoid the issue of ordinal data. With respect to VLSM, the PLS family abstracts sets of specific deficits into their linear combinations, similar to PCA: ensuring the interpretability of these combinations may further require constraining or post-processing results, e.g., with regularizations or sparsification. Our work maintains a direct reference to both spatial correlations between lesioned voxels as well as behavioral correlations of symptoms. Concerning stroke studies, the original view of behavioral deficit networks is partly shared by trajectory profile clustering [25] and recovery Latent Class Analysis [16]. Outside of stroke, sub-typing neurological diseases based on graph theory is explored in [26] for Alzheimer’s, in an unimodal setting where cortical thickness measure profiles are used to evaluate subject similarity. The similarity network is clustered with the Louvain method [27]. In the present study, based on behavioral tests only, spectral clustering [28] is preferred, for the sake of stability [29]. In conclusion, the novelty of our work is in embracing ordinal data, applying spectral clustering to behavioral deficit profiles, and thus performing a data-driven selection of the many-to-many associations between lesioned voxels and behavioral symptoms.

## Materials and Methods

### Data

The NIHSS data consist of 15 items describing the health state, abilities, and cognitive functions of first-stroke ischemic patients from Padua University Hospital and from Washington University in St. Louis. Some items are based on exterior observations that assess damage in specific areas of the brain, while other items test patients with more complex tasks, possibly involving disparate brain regions. NIHSS scores here refer to the acute phase. The average time between the stroke event and the NIHSS assessment for the $n=189$ ischemic subjects in Padua is $4.7 \pm 2.8$ days, while for the $n=119$ ischemic subjects in St. Louis, it is $12.8 \pm 4.6$ days. The average age instead is $68.9 \pm 15.0$ years for Padua and $55.3 \pm 11.7$ years for St. Louis. A brief summary of statistics of the NIHSS items for the 308 ischemic subjects, are reported in the supplementary material Appendix 1. Statistics and clustering are computed and performed on the subset of subjects from both hospitals, with total NIHSS $>0$ and with available brain scans, resulting in $n=172$ patients selected. The initial selection assumes that a total NIHSS score of 0 results from lesions that do not affect function in a measurable way, while the latter selection enables symptoms-to-lesions mapping that would otherwise be impossible. The Level Of Consciousness (LOC)-Vigilance item is removed and the only subject not scoring 0 is filtered out: LOC-Vigilance$>0$ signals that the subject is not conscious and other deficits cannot be assessed, directly leading to missing data and the impossibility of relating lesions to symptoms properly.

**Fig. 1:**
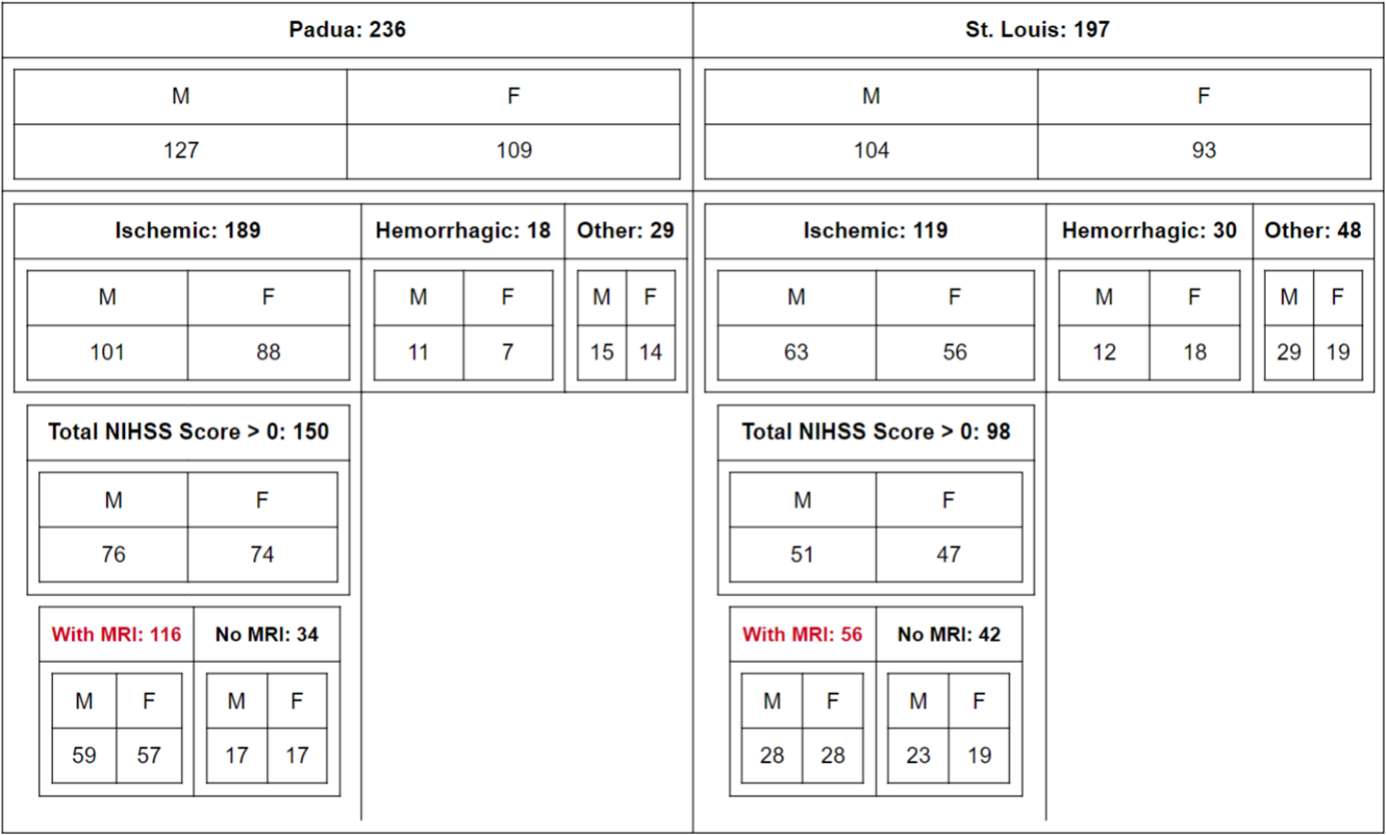
Patient selection workflow. Red marks patients taken into account for clustering. Abbreviations: M, male; F, female

Imaging data come from CT and MRI scans, co-registered in MNI152 standard space [30] at 1 mm$^3$ resolution, with lesions segmented by professionals. The lesion volume is calculated from the lesion masks in the co-registered space; the average lesion volume is $32.71 \pm 47.37$ mL. The population lesion distribution for the 172 patients is reported in Appendix 1. A summarizing scheme of data selection is shown in Fig. 1.

### Correlations and Distances

Denoting *P* the set of *n* patients, and $X_{n=172,m=14}$ the matrix of NIHSS observations considered, the relationship between NIHSS items across the population is explored with statistics suitable for ordinal data. Deficits co-occurrences are counted irrespective of severity: for each couple of items, the number of patients exhibiting both deficits is counted. Then Spearman’s rank correlation coefficient among items is computed, to measure positive and negative associations of severity. These are all operations of type $X^{T}X_{(m=14,m=14)}$ and only serve as descriptive statistics of the dataset.

The General Distance Measure [31, 32] (GDM) is the proximity measure quantifying how different two patients are, based on their profile composed of 14 NIHSS scores. The GDM is defined as taking inspiration from Kendall’s General Correlation Coefficient, in order to process continuous as well as ordinal values. It is here corrected and simplified in notation as:

1 $$\begin{aligned} d_{ab}=\sum _{j=1}^{m}d_{abj} \quad \text {with} \quad d_{abj}= \frac{1}{2m}-\frac{-\sigma ^{2}_{abj}+\sum _{c=1,c\ne a,b}^{n}\sigma _{acj}\sigma _{bcj}}{2\biggl [ \sum _{j=1}^{m}\sum _{c=1}^{n}\sigma _{acj}^{2}\sum _{j=1}^{m}\sum _{c=1}^{n}\sigma _{bcj}^{2}\biggr ]^{\frac{1}{2}}}, \end{aligned}$$

and

2 $$\begin{aligned} \sigma _{abj} = {\left\{ \begin{array}{ll} 1, \text { if } x_{aj} > x_{bj}\\ -1, \text { if } x_{aj} < x_{bj}\\ 0, \text { if } x_{aj} = x_{bj}\\ \end{array}\right. } \end{aligned}$$

with

- $a,b,c = 1, 2,..., n$: indexes of the *n* subjects (stroke patients)
- $j = 1, 2,..., m$: index of the *m* variables (NIHSS items)
- $x_{aj}$: value of the variable *j* for subject *a* (NIHSS score)
- $\sigma _{abj}$: sign of the difference between patient *a* and patient *b* for item *j*
- $d_{abj}$: distance between subjects *a* and *b*, on item *j*.
- $d_{ab}$: distance between subjects *a* and *b*.

Note that the GDM and its complement, the General Similarity Measure (GSM, of value $s_{ab}:= 1-d_{ab}$), depend on the whole cohort observations *X* for each pairwise computation. These are operations of type $XX^{T}_{(n=172,n=172)}$, thus computing similarities between patients can be seen as the dual problem with respect to that of computing correlations between variables. The matrix of all pairwise relationships is symmetric, describing an undirected weighted graph: it is hence called the adjacency matrix $W_{(n=172,n=172)}$ for the graph *G*(*P*, *W*), with *n* nodes referring to the patients and $n(n-1)/2$ weighted edges referring to the GSM of all pairs of patients. For simplicity, *W* will hereon denote the matrix or the network, depending on context.

### Repeated Spectral Clustering

Repeated Spectral Clustering (RSC) is a novel consensus clustering approach, defined in this paper based on spectral clustering, and aimed at dealing with the variability of results that stems from random initializations. Spectral clustering is the name for techniques based on the spectrum of the adjacency matrix’s (or graph’s) Laplacian [28]. Further arguments for using spectral clustering instead of other common techniques, such as hierarchical clustering and *k*-means, can be found in supplementary material Appendix 2. Spectral clustering consists of two steps: spectral embedding, and clustering in the embedding space. The spectral embedding step yields Euclidean coordinates for the network nodes, in the space defined by the eigenvectors of the graph Laplacian. The actual clustering step can be any algorithm working in Euclidean spaces, typically *k*-means in embedding space [33], as in the present case. RSC is also a consensus clustering technique, with two distinct phases of spectral clustering. In the first phase, there is evidence accumulation: the results of *N* different spectral clustering runs on *W* are recorded in the $(n \times n)$ consensus matrix *C*. In the second phase, *C* itself undergoes spectral clustering [34]. RSC is defined by the use of $L_{sym}$ spectral clustering [33] for both phases, whereas other consensus clustering methods change algorithm between the two phases, or change algorithm and/or data subset in the *N* runs of the first phase. While the entries of *W* measure the similarities between any two subjects, the corresponding entries of *C* count how many times those any two subjects are clustered together over the *N* runs of the evidence accumulation phase [35] (clustering co-occurrence, from now on co-clustering). The more times two subjects are co-clustered, the more evidence those subjects are actually similar and co-members of a statistically stable cluster. Matrix *C* too induces a weighted undirected graph and its spectral properties and visual aspect highlight how the subject nodes are better separable than they are in *W*. Notably, while data partitions based on *W* may vary widely due to random initializations, both the entries of *C* and the resulting partitions are remarkably stable. Defined in this way, RSC unifies the ability to find clusters in spectral embedding with the robustness of results regarding centroids initialization, thanks to the evidence accumulation stage. As with other algorithms akin to *k*-means, the number of clusters *k* is set a priori or heuristically: here, the methodology relies on the spectral properties of *C* as a function of *k*. Overall, RSC can be interpreted as a way to extract the clustering signal *C* in the noisy *W*, averaging multiple measurements. The modules of RSC are described in Algorithms 1, 2, and 3 for spectral embedding, clustering, and the whole RSC, respectively. Appendix 3 provides a deeper introduction to spectral clustering, an analysis of how the number of random initializations impacts and stabilizes the final results, and detailed arguments for the better separability of *C* with respect to *W*.

**Algorithm 1:**
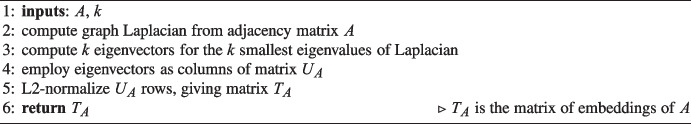
Spectral embedding.

**Algorithm 2:**
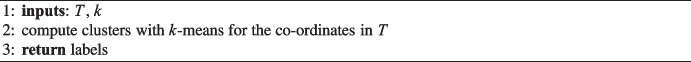
Clustering.

**Algorithm 3:**
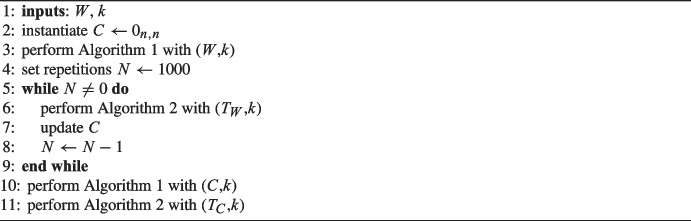
Repeated spectral clustering.

### Voxel-wise Analysis

The application of RSC to the affinity matrix obtained from the GDM pairwise similarities leads to the isolation of clusters. In order to evaluate how a cluster is different from the others, lesion density maps can be visually compared in Fig. 4. Each map is obtained by averaging the binary lesion masks in the respective cluster: thus, each voxel’s intensity represents the normalized frequency (scaled between 0 and 1) of lesions occurring in that voxel within the cluster. In other words, it shows the proportion of patients in the cluster with a lesion in that location. To quantitatively test how significant are differences between clusters, we further perform a voxel-wise analysis. Since the objective of the clustering algorithm is to separate clusters in the space related to NIHSS items, statistical tests on the separation in these same variables would lead to inflated p-values. For this reason, the statistical analysis focuses on voxels that are lesioned with significantly different frequencies across clusters.

The analysis conducted is composed of the following steps:

1. The comparisons are done in a one vs all-but-one way; the null-hypothesis $H_0$ affirms that the lesion distribution in one cluster is not different from the lesion distribution of all the remaining patients.
2. From the $182\times 218\times 182$ available voxels in MNI-152 template, we select those that are lesioned at least 8 times, which corresponds to the 5% of the patients. This is both for a statistical reason, since these voxels carry a greater effect size, as well as a computational reason, since this selection considers only 241163 voxels.
3. The test performed is the difference between proportions with pooled variance [36]:
  3 $$\begin{aligned} t_v=\frac{|\frac{v_i^+}{n_i} -\frac{v^+-v_i^+}{n-n_i}|}{\sqrt{\frac{v^+}{n} (1-\frac{v^+}{n})(\frac{1}{n_i} +\frac{1}{n-n_i})}} \end{aligned}$$
  where
  $n_i$: number of patients in cluster *i*.
  $v_i^+$: number of times voxel *v*, is lesioned ($+$) considering patients in cluster *i*.
  *n*: number of patients (172).
  $v^+$: number of times voxel *v*, is lesioned ($+$) considering all the patients.
4. A permutational approach is used, where patients’ cluster assignment is randomly shuffled; for each selected voxel the statistic is computed 5000 times. In this way the distribution of the statistic $t_v$ can be built under $H_0$, where there is random assignment of patients’ lesions to clusters.
5. Due to the large number of hypotheses under test (1 for each voxel), a multiple-comparison correction is needed. Both Bonferroni and Holm’s [37] methods are largely conservative, so a multistep-maxT (Westfall & Young [38]) procedure is used instead. This allows a Family Wise Error Rate (FWER) control at $\alpha =0.01$.

Since RSC is performed with *k*=5, the presented methodology is applied five times. These other five comparisons are not taken into account by the max-T algorithm; consequently, a Bonferroni correction is applied. Therefore, the results are said to be overall corrected, with FWER at $\alpha =0.05$.

### Open-source Software Tools

Manual segmentations and template normalizations preliminary [10] for this work were done respectively with ITK-snap [39] and ANTs [40]. Filtering data, statistical analyses, clustering, lesion frequency map computation, and visualization are all performed with the Python 3 programming language, and its libraries numpy, pandas, scipy, scikit-learn, nibabel, and nilearn. Voxel-wise analyses are conducted with the R programming language, and its libraries foreach, doFuture, utils, scales, reticulate. The multi-step maxT correction is done using the code available at https://github.com/livioivil/r41sqrt10. In order to allow the usage of the presented analytic workflow by the scientific community on different datasets, code is available at https://github.com/MedMaxLab/nihss_clustering; data will be available upon reasonable request to the authors.

## Results

### Correlations

**Fig. 2:**
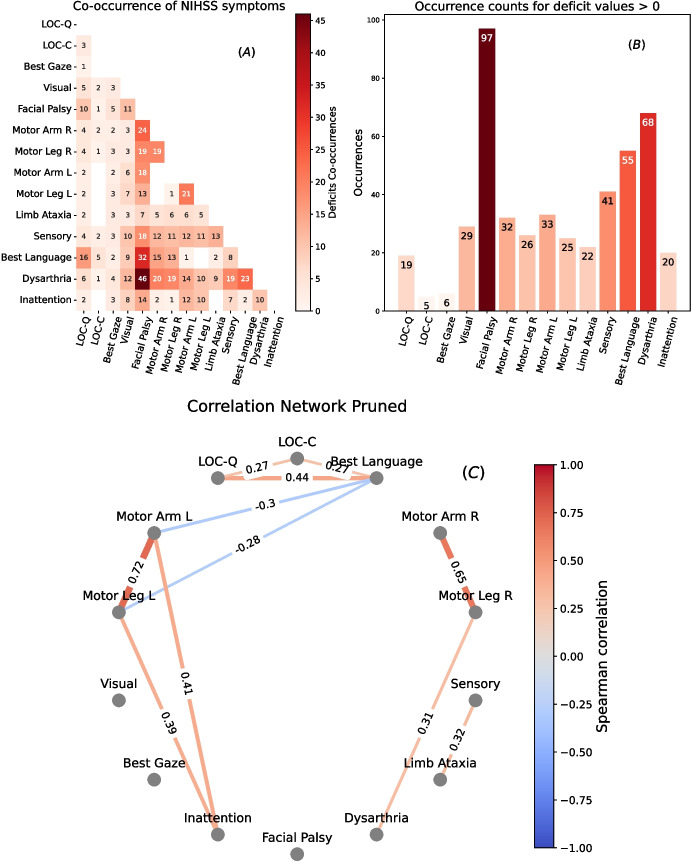
**A** Table visualization of deficits co-occurrences of every item. Intense red for higher number of deficit co-occurrences. Empty blocks represent zero deficit co-occurrences. **B** Bar graph indicating occurrences for every item deficits. **C** Graph visualization of correlation of item deficits severity. Only significant correlations ($\alpha <0.05$, Benjamini-Hochberg FDR correction) are visualized. Abbreviations: LOC, Level Of Consciousness; LOC-C, LOC-Commands; LOC-Q, LOC-Questions; L, left; R, right. Color and thickness of the lines represent the value of Spearman’s $\rho$

The first set of results illustrates the deficits co-occurrences, i.e., the contemporary presence regardless of severity scored, and the rank correlations of deficits across subjects, accounting for their severity (scores ranging from 0–2 to 0–4 for items in the NIHSS). Figure 2A shows the deficits co-occurrence matrix.

**Fig. 3:**
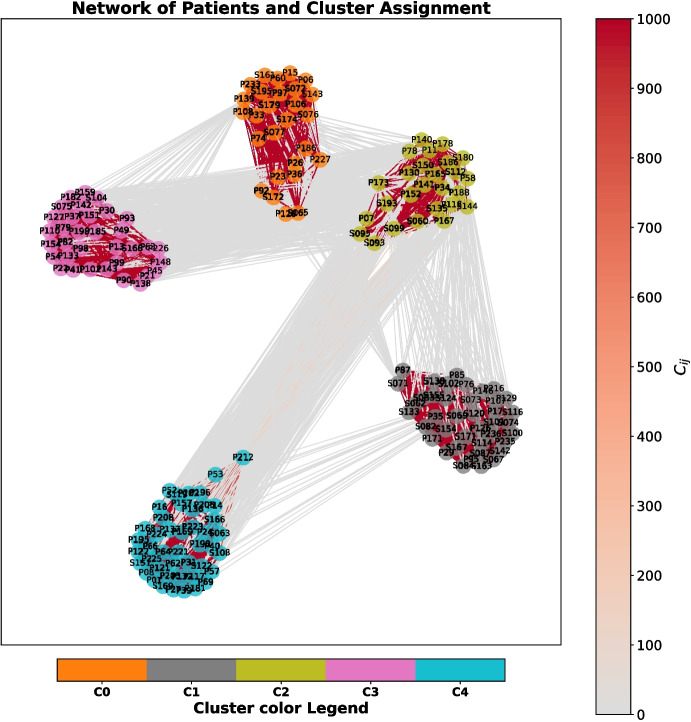
Graph visualization of the clustering co-occurrence matrix *C*. Nodes represent patients, while colors identify clusters. Darker red color links patients frequently clustered together

Facial Palsy and Dysarthria co-occur most frequently, in 46 patients. They also frequently co-occur with language deficits, with 32 patients exhibiting Facial Palsy and 23 showing Dysarthria. Both are linked with motor and sensory impairments on both sides of the body. Language deficits, especially “Best Language,” are often associated with right-sided motor deficits, sensory issues, and visual impairments. Inattention typically accompanies left-sided motor deficits, sensory problems, and visual impairments. Level Of Consciousness-Commands (LOC-C, ability to understand and execute a simple motor command) and “Best Gaze” deficits have fewer associations with other deficits. Figure 2B shows the single deficit occurrence, as a reference to evaluate co-occurrences according to each specific deficit frequency. Facial Palsy and Dysarthria are the most common symptoms in general.

Figure 2C shows only significant correlations after Benjamini-Hochberg FDR correction for multiple comparisons ($\alpha =0.05$) on permutation-based p-values ($10^4$ permutations). For $m=14$ variables, $m(m-1)/2=91$ pairwise correlations are available. Of these 91 correlations, only 11 are significant after correction. The network plot reveals a strong correlation between arm and leg Motor deficits on both sides. “Best Language” shows a significant correlation with LOC-Q, a negative correlation with left Motor deficits (being often co-occurring with right Motor deficits). Left Motor deficits correlate significantly with Inattention, which in turn correlates with Visual deficits. Interestingly, Sensory deficits correlate significantly with Limb Ataxia.

**Fig. 4:**
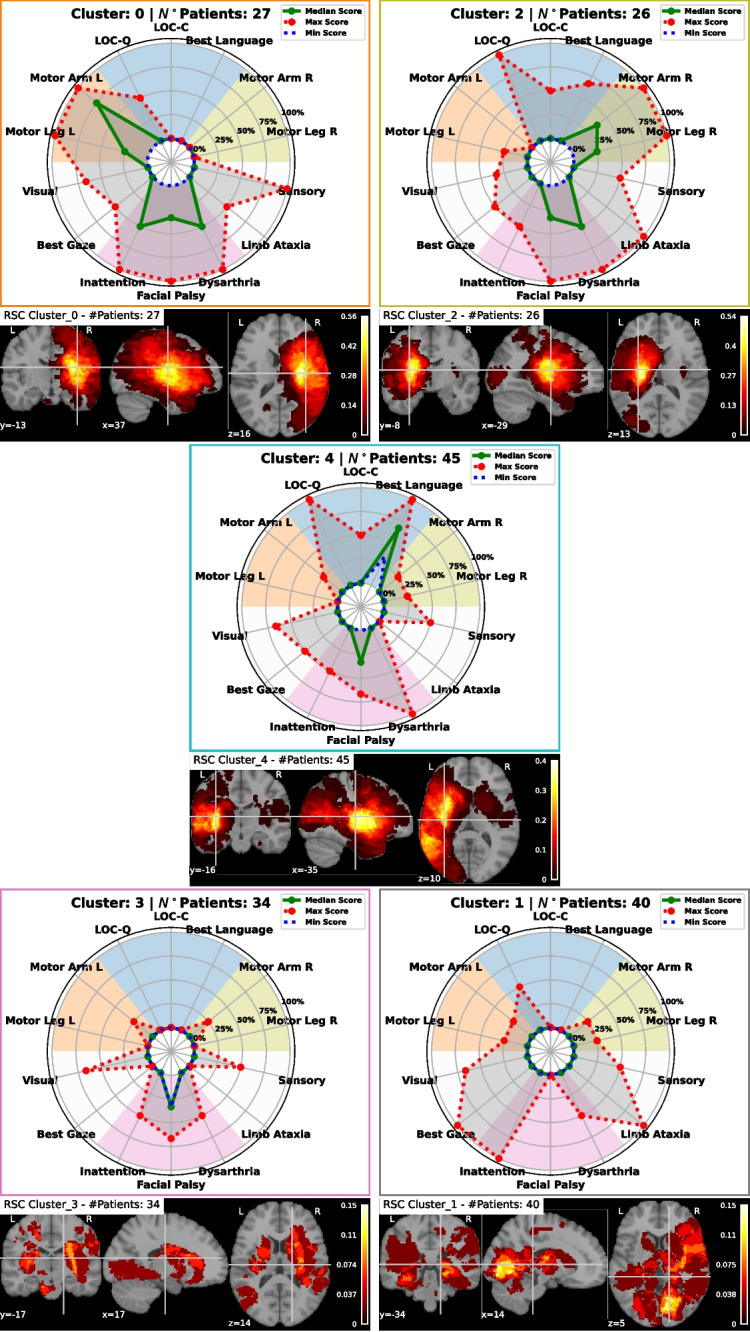
Radar charts and lesion heat maps for the clusters obtained from RSC. In the radar plots the lines represent (in blue) the minimum, (in green) the median, and (in red) the maximum of the NIHSS values inside the cluster. In the heatmaps, brighter colors indicate voxels lesioned by more subjects

In summary, the deficits co-occurrence patterns and correlation network show that arm and leg Motor deficits positively covary on the same side of the body, and negatively covary on opposite sides. Language deficits may occur alone or with right Motor deficits, while Inattention covaries with left Motor deficits. Facial Palsy and Dysarthria are the most common deficits, and often co-occur with other deficits, regardless of side. However, when correcting for significance, they do not privilege any correlation, except for Dysarthria and Right Leg.

### Repeated Spectral Clustering

Applying RSC allows to identify 5 main clusters of patients, shown in Fig. 3. The $k=5$ process shows the sharpest change in the spectrum of the matrix *C*, as discussed in Appendix 3. The minimum, median, and maximum NIHSS profiles for the 5 clusters and the corresponding group average MRI lesion anatomy are visualized in Fig. 4. The median profile is the most informative characteristic to distinguish clusters, with the minimum profile adding nuance especially when different from 0 or equal to the median (e.g., Facial Palsy in Cluster 3). Maximum scores describe the eventual outliers. Given the ordinal nature of the scale, standard deviations and averages are not computed and reported. The Fruchterman-Reingold force-directed algorithm [41] is used for visualization so that patients who are more similar appear closer in the graph (see Fig. 3).

Cluster 0 includes left arm-leg Motor deficits, Inattention, Facial Palsy, and Dysarthria. In the most severe cases, Visual, “Best Gaze,” and Sensory deficits can also occur (see the difference between median and max deficit). The lesions localize to the vascular territory of the right middle cerebral artery (MCA). The cluster average lesion volume is $64.5 \pm 82.4$ mL.

Cluster 1 shows a high degree of deficit variability with a preference for Inattention, Visual deficits, and gaze disorders. It primarily involves the territories of the posterior cerebral artery bilaterally (both deep and superficial branches), and only partially the right MCA (bilateral posterior cerebral artery, PCA). The cluster average lesion volume is $19.2 \pm 23.2$ mL.

Cluster 2 includes right arm-leg Motor deficits, Facial Palsy and Dysarthria. The lesions are roughly localized to the deep left MCA branches; in detail, it involves the medial and lateral lenticulostriatal arteries of the M1 branch of the left MCA. The cluster average lesion volume is $30.7 \pm 43.8$ mL.

Cluster 3 shows a relevant presence of Facial Palsy, bilateral upper limb Motor deficits, Dysarthria, and Sensory deficits. It involves the anterior choroidal arteries and penetrating branches of the MCA, Anterior Celebral Artery (ACA), and PCA bilaterally, as well as penetrating branches of the basilar artery (lacunar infarcts). The cluster average lesion volume is $10.4 \pm 16.7$ mL. In comparison to the lesion volume of all the patients of the other clusters, Cluster 3 shows a significant smaller volume (Kruskal-Wallis’ test $H(1)=10.2,~p=.0014$).

Finally, Cluster 4 involves selectively language deficits and Facial Palsy, and in the most severe cases can affect vision, sensation, and gaze. The lesions are localized in the territories of the superficial left MCA involving the cortical branches of the MCA, including the anterior temporal branch and the superior and inferior branches of M2. The cluster average lesion volume is $43.6 \pm 38.0$ mL.

In summary, RSC provides a distribution of symptoms’ clusters confirming the presence of contralateral Motor syndromes, the relative segregation of language deficits from Motor deficits, and the association of Inattention and Sensory deficits with right hemisphere lesions. A study of how these clusters change when including less or more subjects, and filtering by hospital cohort, is described in Appendix 4.

### Voxel-wise Analysis

**Fig. 5:**
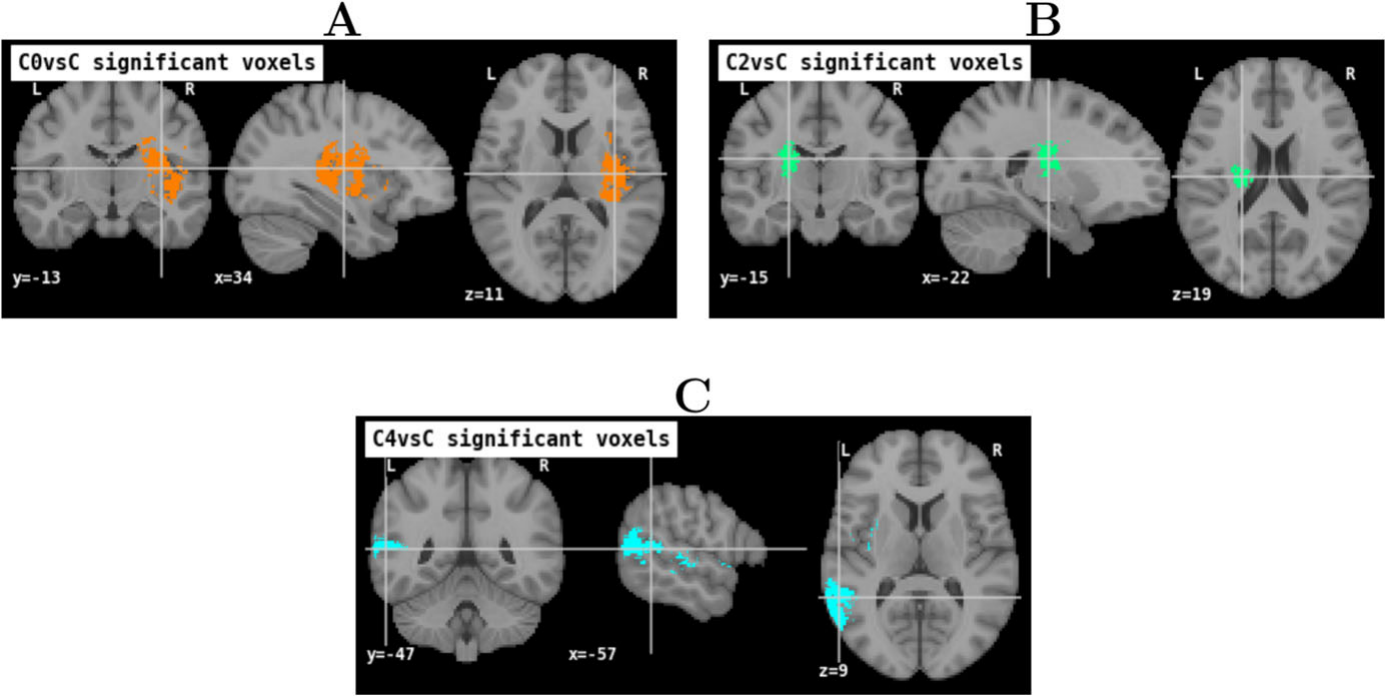
Significant voxels found from the voxel-wise analysis with FWER at $\alpha =0.05$. At the top-left **A** in orange, Cluster 0 (left motor deficits), at the top-right **B** in green, Cluster 2 (right motor deficits) and at the bottom **C** in light blue, Cluster 4 (language deficits)

Applying the voxel-wise analysis allows to find statistically significant distributions of lesions in three clusters: Cluster 0 (left Motor deficits), Cluster 2 (right Motor deficits), and Cluster 4 (language deficits). Significant regions are shown in Fig. 5. The multiple-slices views are shown in the supplementary material Appendix 5. Cluster 1 and Cluster 3 do not present any voxel that is lesioned in a significantly different way with respect to the rest of the cohorts.

Concerning Cluster 0, the orange region in Fig. 5A, is part of the vascular territory of the lenticulostriate arteries of the right MCA. Anatomically, this corresponds to the right corticospinal tract at the level of the corona radiata and the posterior limb of the internal capsule, as well as the posterior insula, globus pallidus, and putamen [42].

For Cluster 2, the green region in Fig. 5B, is part of the vascular territory of the lenticulostriate arteries of the left MCA. Anatomically, this corresponds to the left corticospinal tract at the level of the corona radiata and the posterior limb of the internal capsule.

Finally, for Cluster 4, the light blue regions in Fig. 5C, are within the vascular territory of the inferior trunk of the left M2. Anatomically, this corresponds to the posterior insula, Heschl’s gyrus, superior temporal gyrus (Wernicke’s area), angular gyrus and Geschwind’s area, and arcuate fasciculus [43, 44].

In summary, the patients’ lesions of these three clusters are highly specific with respect to the rest of the patients, and thus characteristic of these clusters. On the contrary, Cluster 1 with its heterogeneous symptoms of mild severity, and Cluster 3 with its Facial Palsy without discernment of lateralization, do not comprise voxels of peculiar significance.

## Discussion

The clustering results obtained from the NIHSS data alone, along with the corresponding imaging outcomes, align with the medical literature. Interestingly, they add anatomical discriminative power to the NIHSS tests, leveraging rank correlations to distinguish similar deficits with different underlying lesions, reflected by different co-occurring deficits. Moreover, the application of RSC to the GDM/GSM measure, and the workflow code are themselves useful results, made available to researchers in biomedical domains.

### The Underlying Structure of the NIHSS

Previous factor analyses of the NIHSS (Lyden et al. [8], Zandieh et al. [7]) identified two main factors, related to the left and right hemispheres, that can explain the majority of variance of behavioral impairment following stroke. While these results assess the conformity of the NIHSS to cerebral hemispheric lateralization, hence fundamentally validating the scale in its clinical setting, they offer limited acute stroke-phase insights for clinicians.

Here, using an unsupervised machine learning approach based on the NIHSS scores, we identify a finer-grained structure of post-stroke impairment. RSC, in addition to left and right hemispheric clusters, identifies a group of co-occurring deficits (i.e., facial palsy, bilateral upper limb motor deficits, dysarthria, and sensory deficits) and anatomical correlates (i.e., bilateral basal ganglia, internal capsules) that may be associated with lacunar syndromes. This clinical entity reflects a specific etiological mechanism (i.e., arteriolosclerosis [45]) that guides the diagnostic and therapeutic algorithms for these patients. Moreover, RSC identifies numerous left hemisphere clusters, possibly due to NIHSS’s higher sensitivity to left lesions [46]. In addition, the limb ataxia subtest was scored in supra-, infra-tentorial, and in bilateral lesions. While this could be related to the rare occurrence of parietal or lacunar ataxia, this could also be associated with the described unreliability of this subtest. This is in line with previous studies that have shown low inter-rater reliability of this subtest, proposing modified versions of the scale for clinical trials (see the modified NIHSS, or mNIHSS [47, 48]).

Notably, RSC seems to identify a cluster (Cluster 1) with highly heterogeneous patients (see Fig. 4, radarplot in gray). This cluster may be interpreted as patients without clear behavioral similarities (i.e., “leftovers”) according to the NIHSS, reflecting the necessity to integrate this test with additional hyperacute clinical evaluations, including cognitive and mood assessments, as shown by previous work [9, 49].

Finally, our voxel-wise analysis identifies a statistically significant set of lesions for Cluster 0 (left Motor deficits), Cluster 2 (right Motor deficits), and Cluster 4 (language deficits). In particular, motor deficits are significantly associated with corticospinal tract damage, while language deficits with damage to the arcuate fasciculus and Wernicke’s area. Interestingly, the described regions correspond precisely to anatomical substrates with a well-established prognostic role for recovery [50–54].

Future work, using the same validated methodological approach, could assess the longitudinal evolution and anatomical specificity of the identified clusters to better characterize post-stroke recovery trajectories.

### Unsupervised Learning Approaches in Stroke

This work introduces a new perspective on stroke cohort data analysis. Patients are represented in a network where they are linked by similarity, then clustered.

Overall, the traditional analyses based on PCA-like methods focus on problems of the form $X^{T}X$ when *X* is a matrix of *n* observations (subjects) on *m* variables; for VLSM, PLS and CCA studies of neuroimages and behavioral measures, the matrix product is $X^{T}Y$ when *Y* has the same *n* subjects observed, and there are $m_{X}$ variables in the set of features $F_X$ (e.g., behavioral scores), and $m_{Y}$ variables in the set of features $F_Y$ (e.g., brain voxels). In contrast, the perspective here focuses on patient profiles, patient-to-patient and patient-to-group relations, and group-specific associations of deficits and lesions, addressing the matrix product $XX^T$. This explains the complementarity of this approach with existing literature. Data points are clustered on the set of features $F_X$ (here, NIHSS sub-scores), and cluster statistics are evaluated on the feature set $F_Y$ (binary masks of brain lesions in CT and MRI scans). The process leverages the multimodality of both $F_X$ and $F_Y$, translating to different domains. It is also important to underline how the set of features $F_X$ can be expanded, e.g., concatenating measures from several neuropsychological test batteries, or even physiological records. The pipeline here presented can be applied without changes to these settings, with both ordinal and continuous variables, as RSC clustering concerns only the similarity network. Continuous variables could also undergo dimensionality reduction before computing GDM values if considering how the computation scales with the number of variables. In the current setting, starting from the first principles, the suitability of the GDM for ordinal data ($F_X$) is demonstrated and used to construct the similarity network of NIHSS score profiles. The most common clustering distances include Euclidean, cosine, Manhattan, Mahalanobis, and Pearson distances, as noted in [55]. Differences in pairwise similarity distributions can be found in Appendix 6. Using the cosine distance would result in most patients being orthogonal, thus maximally different. However, this property is not desirable, as scoring 1 in two different items does not make two subjects maximally different, one argument being that they are both close to the healthy case where all NIHSS scores are zero. Euclidean distance is unsuitable and not theoretically motivated for the NIHSS data space, since it is a lattice space, where different distances on different axes are arguably not proportional (anisotropy and inconsistency of scale). Mahalanobis and Pearson distances are also not suitable for NIHSS data, since they assume the validity of standardization operations (mean-centering and variance-scaling) on the data, which are intrinsically skewed. Manhattan distance works well with interval data. However, the GDM is still superior to Manhattan in these respects: because it accounts for the variability in each item, intensifying the impact of pairwise differences the less entropy is found in an item; because it does not depend on the magnitude of the differences, making no assumptions on the scales of different items, thus shrinking the distribution of similarities.

NIHSS items difference between two subjects conveys the difference in gravity of the condition, but the gravity itself is not linear, as suggested instead by the use of single digits in the NIHSS scale. This motivates the use of GDM for such data as theoretically sound, with parsimonious assumptions, and pushing toward a more conservative position with respect to patient conditions, compared to the differences in type of symptoms. The GDM-based similarity network is clustered using the unsupervised machine learning technique of Repeated Spectral Clustering.

RSC unifies consensus and spectral clustering in a simple frame, taking advantage of the random initializations of *k*-means to derive robustness, as evidenced by the eigenvalue changes in *W*’s and *C*’s respective graph Laplacians (see Appendix 3). To the best of our knowledge, this is the first instance using the normalized symmetric Laplacian algorithm [33] both in the “ensemble” of random initializations constituting evidence accumulation, and as the consensus function aggregating them in a final result, requiring a single *k* choice as in classical *k*-means, supported by the spectral analysis of *W* and *C*. For related algorithms, see  [56] and  [34]. The pros of RSC comprise its statistical robustness, the interpretability of the eigenvalue gaps in the choice of *k*, and its applicability to any type of similarity measure and consequent similarity graph. While a single step of spectral clustering on *W* may yield a different patient division depending on random initialization, the results from the *C* matrix are identical across re-tests. The number of close-to-0-valued eigenvalues, or equivalently the ranking of the max spectral gap of the matrices *W* and *C* indicates the number of separate components in the respective graphs, which suggests the best choice for *k*. For different *k*, the eigenvalues of $C_k$ will change and reflect the “denoising” value of repeated clustering: if the *k*-th spectral gap of $C_k$ has the highest increase over the *k*-th spectral gap of *W*, RSC is interpreted as uncovering the greatest evidence for a *k* component structure hidden in *W*. The cons of RSC are those distinctive of clustering approaches, apart from the computation requirements. Clustering techniques are unsupervised methods with no available ground truth and require the availability of external labels, measures, and the practitioners’ domain knowledge in order to assess statistical robustness and significance levels of results, apart from the internal measures each technique directly or indirectly optimizes. Concerning computational requirements, the researcher can evaluate the need and added value of RSC on a case-by-case basis. We suggest first checking both the spectrum of *W* and the robustness of results over $N\sim 10$ trials of spectral clustering with *W*. If the configurations yielded are several, noisy, or incoherent based on domain knowledge, and if the spectrum has an uninformative profile, it might be the case for RSC clustering with a larger *N*, in line with available computational resources.

Future work would extend the pipeline acknowledging its limitations and enabling its application to larger stroke patient cohorts, more extensive symptom records, and other multimodal biomedical data collections, regardless of prediction targets. While the GDM’s flexibility for ordinal and continuous data is valuable, traditional preprocessing steps could support other proximity measures such as Hamming, Manhattan, and cosine distances, without hindering RSC applicability. RSC could be improved by considering geometric constraints in spectral embedding and incorporating soft partitions and probabilistic cluster attributions, currently implied by the consensus matrix. Extending these tools with a focus on interpretability can provide valuable support systems in research studies.

## Conclusions

In recent years, machine learning and data science have advanced significantly, offering new prospects across many domains. The medical field’s complexity, with its numerous variables and statistical properties, makes it difficult to create data-driven mathematical models of diseases from healthcare data. In this paper, for the first time, we address stroke patient clustering, presenting a novel unsupervised data analysis pipeline tailored to the properties of health scales, and applied to a leading global cause of death and disability. Many studies have analyzed patient behavior and lesion locations, but they often rely on assumptions that can limit their validity, such as treating item modalities as numerical data and using linear models. This research complements existing viewpoints by following a new and precise methodological path and introducing new key elements. The methods presented are specifically tailored to address intrinsic problems of biomedical data. They focus on the thorough treatment of ordinal variables and avoid restrictive, distorting assumptions through the use of GDM and model-free lesion mapping. Additionally, these methods leverage an innovative approach based on evidence accumulation clustering.

The lesion maps show anatomical separation based on cluster membership, aligned with the expected localization of deficits, despite being based solely on the clustering of behavioral data. They describe a detailed structure of neurological syndromes that are in line with topographical and etiological features of acute stroke.

The novel and open source workflow here provided is flexible and of high methodological value, ready for extensive adaptations to multimodal biomedical data in other domains, whenever unsupervised phenotypizing of obervations is difficult but potentially enriching, and whenever clinical healthcare data comprises ordinal scales.

## Figures and Tables

**Fig. 6:**
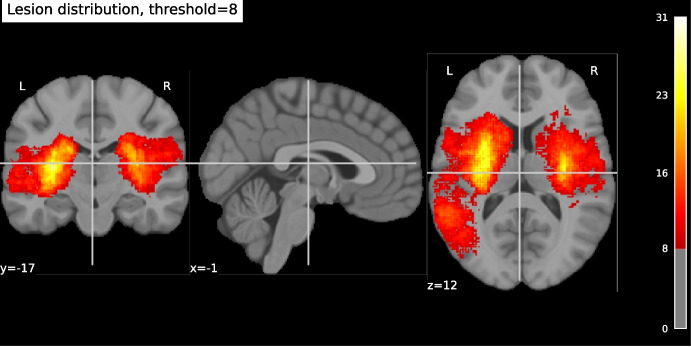
Lesion heat-map for the 172 ischemic patients used for clustering. Voxels lesioned in less than 8 subjects are omitted, for statistical reasons expressed in Section 2.4

**Fig. 7:**
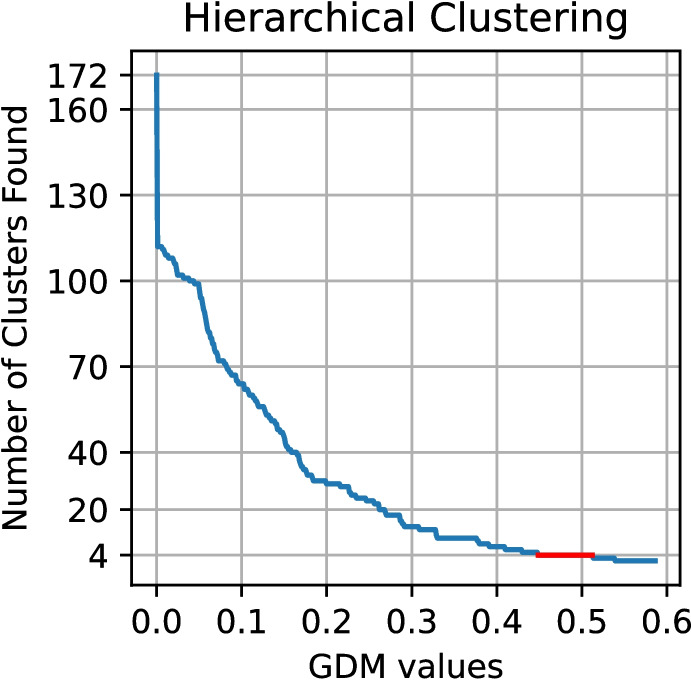
Number of clusters found by the hierarchical clustering versus the distance values. In red is shown the longest interval corresponding to 4 clusters

**Fig. 8:**
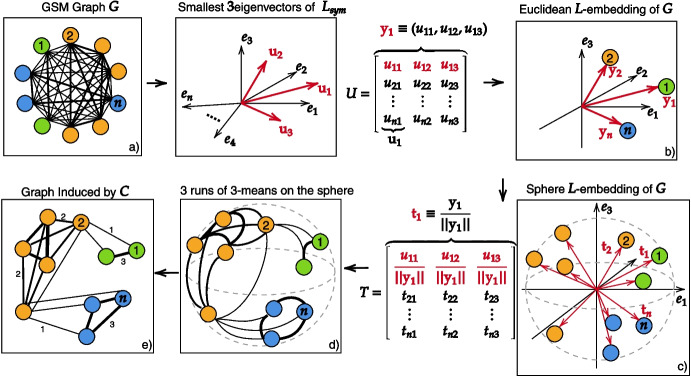
Graphical summary of how spectral clustering works in the case of $k=3$. Starting from **a**, the GSM matrix visualized as a graph, **b** then the matrix *U* is computed with its respective Euclidean embedding. **c**Then, the points are projected to the unit sphere and **d** the *k*-means is run. **e** The *k*-means is repeated *N* times giving the final co-clustering matrix *C* and the corresponding graph

**Fig. 9:**
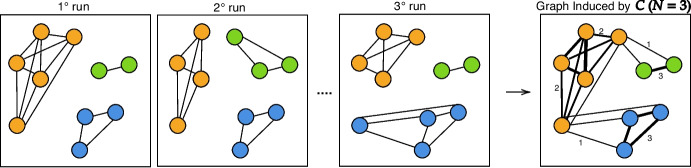
Toy example of how the *C* matrix is built. Here, different data points are clustered together differently for each run of the *k*-means. At the end the *C* matrix is obtained by counting the number of times a link is present between two nodes over *N* runs

**Fig. 10:**
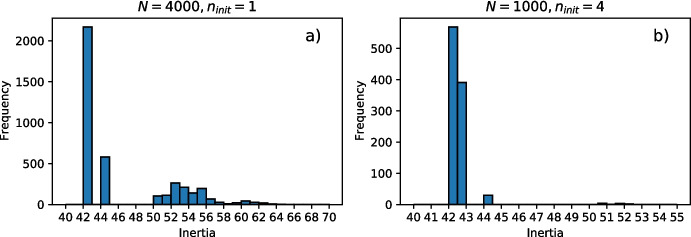
Comparison between histograms of the inertia in the case $n_{init}=1$ (**a**) and $n_{init}=4$ (**b**). The total number of runs is thus fixed to $N*n_{init}=4000$

**Fig. 11:**
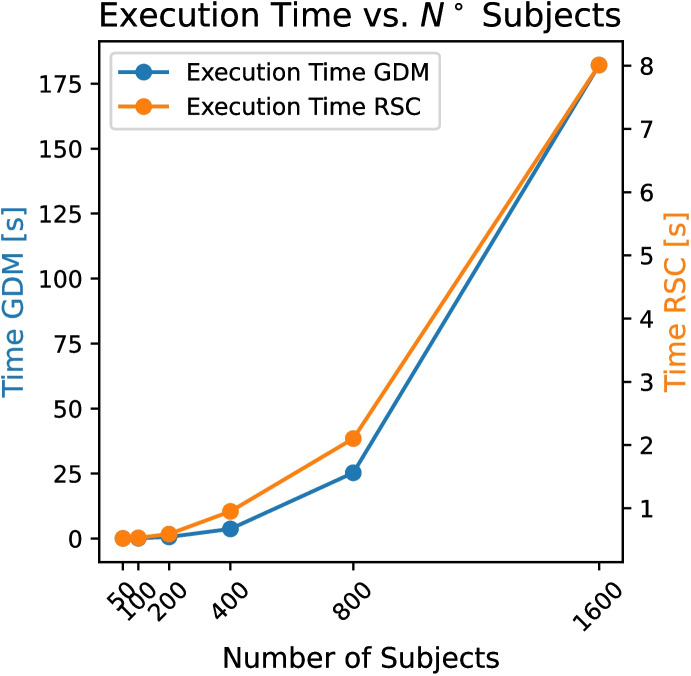
Computational time required by the proposed pipeline. In blue, the time required by the GDM (matrix of distances). In orange, the time required by the RSC (clustering algorithm)

**Fig. 12:**
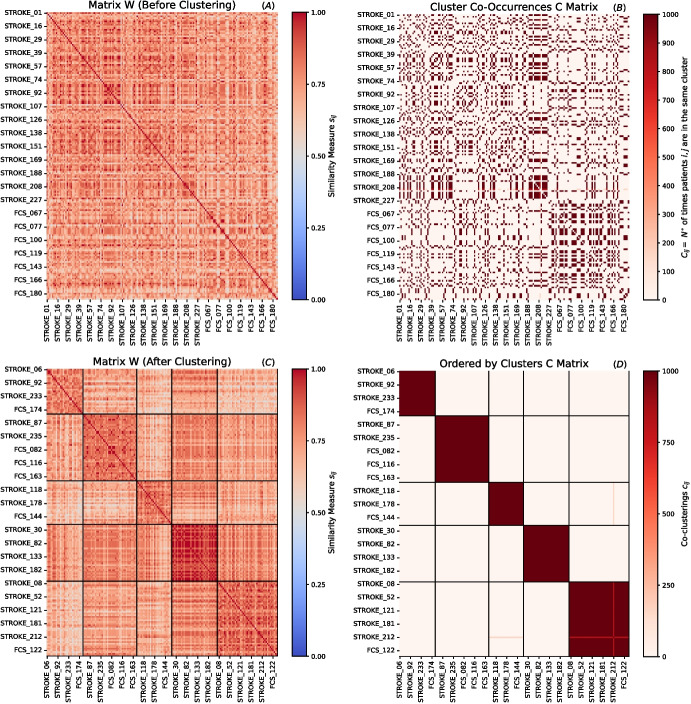
Comparison of matrix *W* and matrix *C*. **A** The similarity matrix *W* for the 172 patients considered for the clustering analysis. **B** The clustering co-occurrence matrix *C*. **C** The same as **A**, but subjects are grouped by cluster assignment; here the matrix is in its block diagonal form. The clusters were not visible in **A**; the allocation of patients to clusters acts as a row and column permutation, making the clusters visible. **D** The same as **B**, but the subjects are grouped by cluster assignment; here, the clusters come from the population under analysis. In **A** and **C**, red colors indicate a higher value of similarity ($s_{ij}$) between two patients. In **B** and **D**, red colors indicate a higher value of clustering co-occurrence ($c_{ij}$) between two patients

**Fig. 13:**
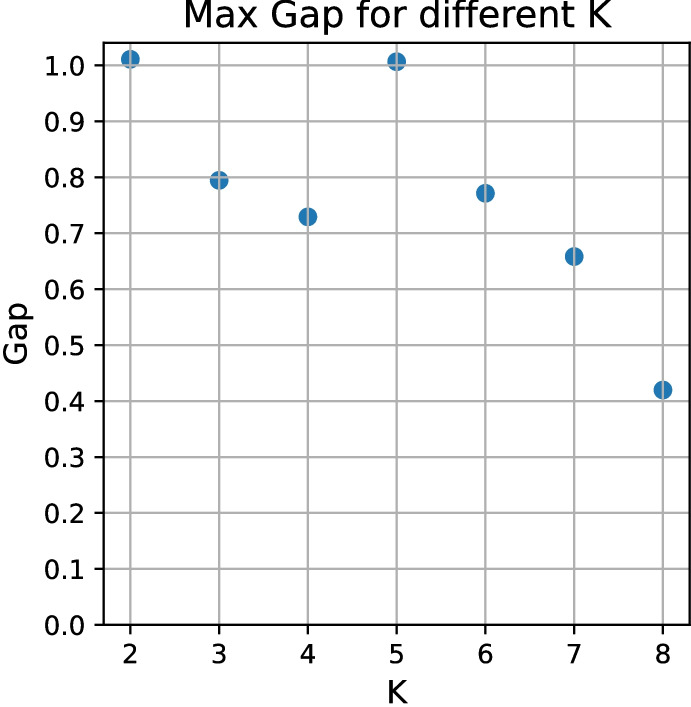
Spectral-gaps obtained from matrix *C* for various *k*, obtained by the RSC procedure applied to the 172 selected patients

**Fig. 14:**
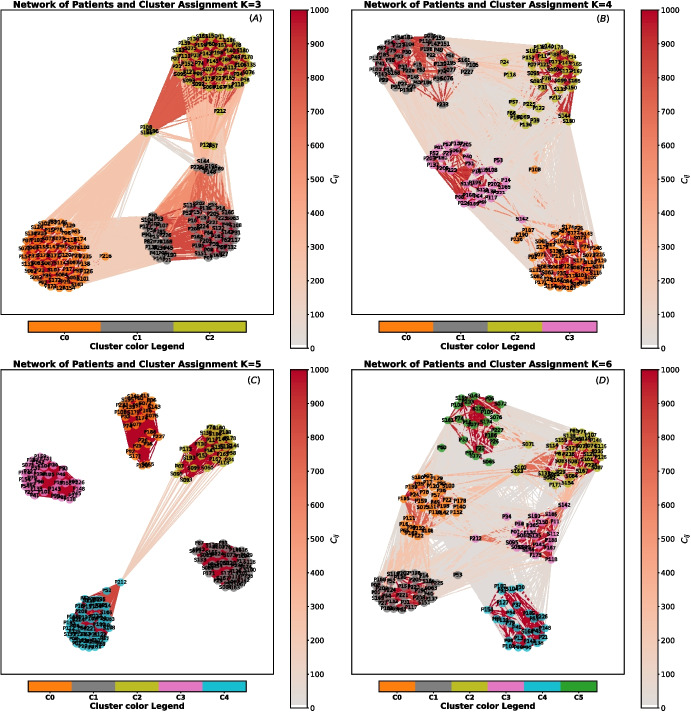
Effects of different *k* on the graphs induced by the matrix *C*. For visualization purposed, edges less then 1% of the repetitions ($N=1000$) have been removed, as it can be noted in the colorbar. Darker red color links patients frequently clustered together. Nodes represent patients, while colors identify clusters. In **A**, clusters obtained with $k=3$; in **B**, clusters obtained with $k=4$; in **C**, clusters obtained with $k=5$; and in **D**, clusters obtained with $k=6$

**Fig. 15:**
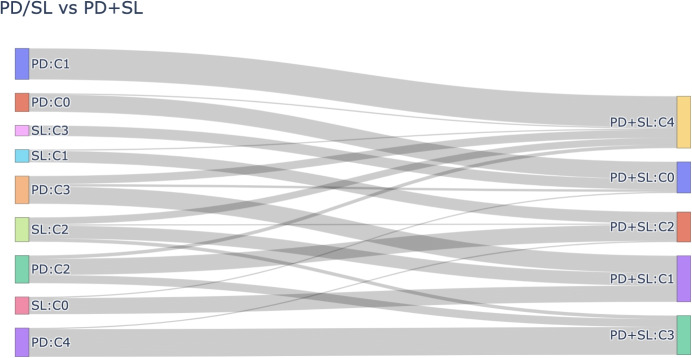
Comparison of patient cluster assignments derived from separate cohorts (PD/SL) versus combined cohorts (PD+SL). PD stands for Padua, SL for St. Louis

**Fig. 16:**
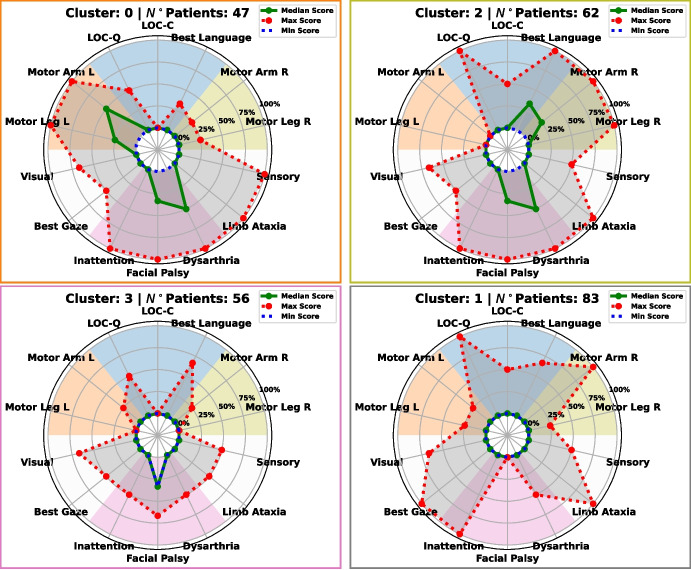
Radar charts for the clusters obtained from RSC, considering the 248 ischemic patients from both cohorts, with NIHSS$>0$. In the radar plots the lines represent (in blue) the minimum, (in green) the median, and (in red) the maximum of the NIHSS values inside the cluster

**Fig. 17:**
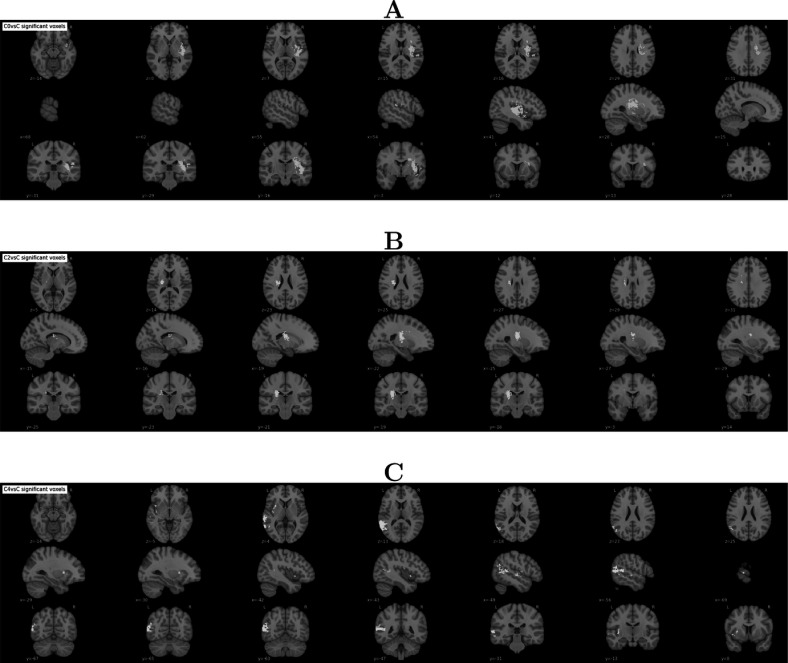
Significant voxels found from the voxel-wise analysis with FWER at $p=0.05$ for different slices. At the top (**A**) in orange, Cluster 0 (left motor deficits), in the middle (**B**) in green, Cluster 2 (right motor deficits) and at the bottom (**C**) in light blue, Cluster 4 (language deficits)

**Fig. 18:**
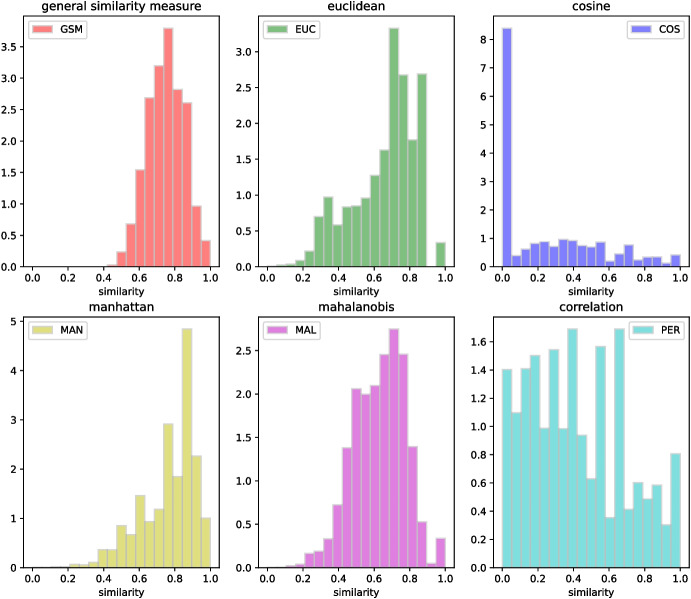
Pair-wise similarities distributions, for six different distances used. GSM stays for General Similarity Measure; EUC stays for Euclidean; MAN stays for Manhattan; MAL stays for Mahalanobis; PER stays for Pearson

## Data Availability

The data that support the findings of this study are not openly available due to reasons of sensitivity and are available from the corresponding author upon reasonable request. Data are located in controlled access data storage at Padova Neuroscience Center.

## Appendix 1. Dataset summary statistic

For all the 308 ischemic patients comprised in the two cohorts, Padua and St. Louis, some summary statistics have been computed, such as mean (M) and standard deviation (SD) of every item, Table 1

**Table 1:** Mean (M) and standard deviation (SD) calculated for every NIHSS item, calculated for all 308 ischemic subjects

NIHSS Item	Padua (189): M$~\pm ~$SD	St. Louis (119): M$~\pm ~$SD
LOC-Q	0.14$~\pm ~$0.46	0.07$~\pm ~$0.31
LOC-C	0.02$~\pm ~$0.13	0.02$~\pm ~$0.13
Best Gaze	0.02$~\pm ~$0.14	0.07$~\pm ~$0.28
Visual	0.16$~\pm ~$0.49	0.28$~\pm ~$0.57
Facial Palsy	0.65$~\pm ~$0.64	0.34$~\pm ~$0.67
Motor Arm R	0.23$~\pm ~$0.68	0.60$~\pm ~$1.32
Motor Leg R	0.16$~\pm ~$0.53	0.35$~\pm ~$1.07
Motor Arm L	0.24$~\pm ~$0.78	0.48$~\pm ~$1.07
Motor Leg L	0.14$~\pm ~$0.56	0.29$~\pm ~$0.88
Limb Ataxia	0.05$~\pm ~$0.25	0.26$~\pm ~$0.54
Sensory	0.10$~\pm ~$0.32	0.40$~\pm ~$0.51
Best Language	0.47$~\pm ~$0.80	0.25$~\pm ~$0.59
Dysarthria	0.33$~\pm ~$0.50	0.38$~\pm ~$0.55
Inattention	0.12$~\pm ~$0.41	0.15$~\pm ~$0.46

The same statistics for the 172 subjects used for clustering, separated by cohorts are reported in Table 2.

**Table 2:** Mean (M) and standard deviation (SD) calculated for every NIHSS item and calculated for the 172 ischemic subjects considered for clustering

NIHSS Item	Padua (116): M$~\pm ~$SD	St. Louis (56): M$~\pm ~$SD
LOC-Q	0.20$~\pm ~$0.55	0.11$~\pm ~$0.41
LOC-C	0.03$~\pm ~$0.16	0.04$~\pm ~$0.19
Best Gaze	0.03$~\pm ~$0.18	0.05$~\pm ~$0.30
Visual	0.18$~\pm ~$0.52	0.36$~\pm ~$0.64
Facial Palsy	0.83$~\pm ~$0.62	0.38$~\pm ~$0.73
Motor Arm R	0.28$~\pm ~$0.77	0.59$~\pm ~$1.33
Motor Leg R	0.20$~\pm ~$0.59	0.46$~\pm ~$1.13
Motor Arm L	0.34$~\pm ~$0.90	0.64$~\pm ~$1.38
Motor Leg L	0.17$~\pm ~$0.64	0.55$~\pm ~$1.17
Limb Ataxia	0.06$~\pm ~$0.24	0.36$~\pm ~$0.64
Sensory	0.14$~\pm ~$0.37	0.48$~\pm ~$0.54
Best Language	0.62$~\pm ~$0.88	0.29$~\pm ~$0.59
Dysarthria	0.41$~\pm ~$0.53	0.45$~\pm ~$0.57
Inattention	0.16$~\pm ~$0.47	0.18$~\pm ~$0.51

The population lesion distribution for these patients having the images is reported in Fig. 6; voxels lesioned by less than 8 patients are not reported.

## Appendix 2. Other Clustering Techniques

*k*-means and hierarchical clustering are two widely used clustering techniques. This section demonstrates why these methods are unsuitable for NIHSS items and suggests alternative approaches.

Since *k* is a hyper-parameter of *k*-means, we experimented with different values ranging from 2 to 9. The raw NIHSS can be input to *k*-means, along with its normalized and standardized versions. The algorithm defaults to using Euclidean distance for this analysis. Concerning the *sklearn* “KMeans” function used, the initialization was set to random, inertia to 10, and the maximum number of iterations to 300, with a random state of 12345. Table 3 reports the number of subjects for each cluster across various normalization combinations and values of *k*.

**Table 3:** Number of subjects, for each cluster, for all the possible combinations of *k* and normalization used

*k*	Standardized	Normalized	Raw
2	157, 15	155, 17	156, 16
3	15, 13, 144	142, 13, 17	16, 13, 143
4	112, 15, 13, 32	111, 17, 13, 31	112, 16, 32, 12
5	93, 19, 13, 32, 15		
6	13, 13, 16, 106, 19, 5		58, 7, 9, 54, 12, 32
7	27, 13, 9, 7, 5, 19, 92	10, 18, 33, 31, 62, 5, 13	38, 21, 10, 6, 17, 67, 13
8	79, 18, 15, 5, 1, 15, 32, 26	37, 12, 10, 62, 23, 5, 10, 13	12, 12, 38, 52, 16, 32, 6, 4
9	27, 18, 15, 5, 1, 15, 13, 26, 52	37, 12, 10, 62, 23, 4, 10, 13, 1	12, 12, 38, 52, 12, 8, 5, 4, 29

In green the configurations with a number of subjects matching the 50–5% criterion

To evaluate cluster quality, consider cluster size: both very large clusters (over 50% of subjects) and very small clusters (under 5% of subjects) are problematic. Large clusters obscure phenotypic differences, while small clusters lack statistical power. Prior hypotheses on the number of clusters and their sizes should be permissive, but finding only 1 cluster or only clusters with 1 subject are degenerate solutions. 50% is the largest intuitive fraction associated with random cluster assignment with a uniform prior in the simplest setting $(k=2)$; similarly, 5% is widely used as a significance threshold in statistics. The same reasoning can be framed in terms of statistical power, as the same 5% threshold was used to select the voxel threshold of 8 patients in Section 2.4. Configurations with fewer than 8 or more than 85 subjects should be thus discarded. With these criteria, the only suitable configurations are normalized data with $k=5$ and $k=6$ and raw data with $k=5$. In these configurations, clusters for Motor Right, Motor Left, and Language deficits were identified.

In all these three configurations the patients in Motor-R are 13, while the patients in Motor-L are respectively 14 for norm data and 16 for raw data; our clusters account better for patients with moderate and low scores in these items, giving 27 and 26 subjects respectively. The patients that are excluded from these three clusters are 74 in our work, while 114 with *k*-means. The main reasons for the observed behavior could be the high-dimensionality of the data, and the spherical-shape of clusters of the *k*-means algorithm.

Hierarchical (agglomerative) clustering is another popular technique used to progressively aggregate data together, based on a distance measure. At each iteration, the subjects below a distance threshold are grouped together. The algorithm results in a dendrogram, a tree-like diagram in which every data point corresponds to the leaves, and branches to track the merging of subjects in larger groups. Varying the distance threshold, the tree gives different numbers of clusters from a maximum equal to the number of patients to only one cluster of all patients. The standard technique to assess the “true” number of clusters is to check the longest interval in terms of distance threshold (GDM), for which the number of clusters does not change. The longer the interval, the stronger the evidence for a structure and number of clusters. In our case, the distance is given by the GDM matrix (thus pre-computed), and the linkage mode was set to complete. Figure 7 shows the number of clusters with respect to the GDM distance, where the optimal *k* was found to be 4. However following the rules before, there are the usual right/left motors and language clusters, but cluster 0 has 96 patients, that is more than half of the dataset.

In conclusion, vanilla *k*-means and hierarchical clustering, are not suited for the NIHSS data of our two cohorts; spectral clustering is the next most commonly used clustering technique and it was used as the base of this work.

## Appendix 3. Spectral Clustering

Spectral clustering is a very powerful clustering technique; however, its geometrical foundation makes it hard to understand at first glance for non-experts in the field. In Fig. 8, it is reported a brief illustrated example of how spectral clustering works.

From the adjacency matrix *W*, built from the similarity measure with $w_{ab}=s_{ab}$, the associated normalized Laplacian matrix $L_{sym}$ is calculated [33]. If the graph of *W* has *n* separate sub-graphs, the Laplacian has *n* eigenvalues equal to 0. Similarly, if the Laplacian has *n* relatively small eigenvalues, the graph of *W* has *n* components far more connected internally than with one another. The eigenvectors associated with the *n* first, the smallest eigenvalues are then used to create the new embedding space, where the data set is projected to. Once the spectral embedding is performed, *k*-means clustering is typically run over the new data set with *k* equal to the number *n* of eigenvectors considered. *k*-means clustering results in a complete, hard partition of the data. The above steps would complete one level of spectral clustering. However, it is well known that *k*-means results depend heavily on the random initialization of the centroids. Consequently, different runs of the algorithm will yield different results and partitions. Ensembling of clustering results, also known as consensus clustering [57], is the process of aggregating different partitions from multiple algorithms into a single, robust one. Evidence Accumulation Clustering [35] is a particular consensus clustering technique. In particular, this technique is based on repeating *N* times the *k*-means algorithm. Through repetitions, evidence accumulates regarding which data samples consistently and reliably cluster together. The so called clustering co-occurrence (or co-clustering) matrix *C* is defined, where the entries $C_{ab}$ are the number of times two subjects *a* and *b* are clustered together over the *N* trials, as shown in Fig. 9.

The matrix *C* describes a new graph, where strong connections represent frequent associations in the same clusters. The default hard partition from *k*-means is lost to a soft partition of connected communities. Since the objective of clustering is still assigning labels to data points, a further clustering method could be applied to the co-clustering matrix *C*, to finally separate the communities in the new co-clustering network. In this work, a further spectral embedding and *k*-means is performed on *C*, given that its spectral properties and separability are enhanced compared to the original adjacency matrix *W*. This idea is analogous to work in [34], where the spectral embedding relies on a different Laplacian form, $L_{rw}$, and the clustering for evidence accumulations are several and different algorithms.

### 3.1 Statistics

Considering the number of times *N* the *k*-means has to be executed, the event *two patients are clustered together* is a dichotomous event; hence $C_{ab}$ follows a Binomial distribution. Since *C* can be written as $C=NP$, $C_{ab}$ are interpreted as mean values, so the error associated with each entry scales as $\frac{1}{\sqrt{N}}$. From statistical reasoning on the error of the variance, the minimum number of trials is, therefore, 200; in this work $N=1000$. Going into the details of the *k*-means function implemented in *scikit-learn*, there is a parameter called $n_{init}$ that indicates how many runs have to be executed to give the final answer, i.e., the labels. The idea behind this is to try different runs and choose that with the smallest inertia, as the best candidate out of the batch. Further details can be seen in the corresponding web page https://scikit-learn.org/stable/. Since in this work, the *k*-means is repeated several times in order to fully exploit the stochasticity of the centroids initialization, in principle $n_{init}=1$. However as can be seen in Fig. 10, moving from $n_{init}=4$ to $n_{init}=1$ gives rise to a bell-like shape probability distribution function, centered in 54; still the main peak of the inertia is in the bins [42, 43].

This indicates that increasing $n_{init}$ a little bit (e.g., 4) is beneficial for having clusters with low inertia and avoid to have noisy-configurations with inertia around 54. However even in the case with $n_{init}=1$ the final labels are equal to the case of $n_{init}=4$.

### 3.2 Computational Benchmark

The time needed for the computation of the General Distance Measure (GDM) matrix and the time needed by the Repeated Spectral Clustering (RSC) algorithms are reported. In detail, a random NIHSS-like dataset with increasing size has been built, and fed to the proposed pipeline.

### 3.3 Matrices *W* and *C*, before and after RSC

This section aims to show the effect of the RSC algorithm, on the matrix of similarities *W*. In detail, the effect of clustering is making this matrix, the most block diagonal as possible. Then the co-clustering matrix *C* is compared to the matrix *W*.

### 3.4 Optimal number of clusters

The way in which *n* is chosen for *W* and *C* relies on the spectra of the normalized Laplacians of the matrices *W*, *C*. As stated above, in classical spectral clustering, *n* null eigenvalues indicate *n* completely separate groups in the data graph, and *n* very small eigenvalues followed by a larger eigenvalue indicate *n* softly distinct groups. So, in the absence of exactly 0-valued eigenvalues, the problem consists in finding a spectral gap, i.e., a large difference between an eigenvalue and all the other quasi-0 predecessors. The *n*-th spectral gap for *W* is the difference between the $n+1$-th and the *n*-th ordered eigenvalues of the Laplacian matrix of *W*. For each value of *k*, repeated clustering of *W* constructs a different matrix $C_k$. The *n*-th spectral gap for $C_k$ is again the difference between the $n+1$-th and the *n*-th ordered eigenvalues of the Laplacian matrix of $C_k$. The max gap criterion would suggest to choose *k* for a given graph so that the *k*-th spectral gap (the difference between $k+1$-th and *k*-th eigenvalues) is maximal. However, the graph of $C_k$ has *k* subcomponents by construction, therefore the maximal spectral gap for $C-K$ is always expected to be the *k*-th. The actual choice must be made among the different max gaps across the matrices $C_k$, for every *k* attempted. Thus domain knowledge and comparisons with the spectrum of *W* must be taken into account to select *k*. The interpretation of the criterion is to choose *k* so that RSC has the highest added value in terms of information.

In Fig. 13 is reported the max gaps of *C* for different *k* trials.

Figure 13 is, therefore, the main tool to evaluate the quality of the cluster solution and thus the main tool to decide the optimal number of clusters. Regarding the results of the main manuscript, the optimal solution found is then $k=5$. There is a priori knowledge of the minimum number of clusters to be found in this domain, which is 3 as the main domains involved in stroke (language, motor right, and motor left): the solution should have at least 3 clusters, and $k=5$ stands out among those clusterings.

Finally, in Fig. 14, the *C* graphs obtained for different *k* are shown, similarly to the case $k=5$ shown in Fig. 3 in the main manuscript. It can be seen that all other solutions ($k={3,4,6}$), showing less separated clusters, respect the optimal solution $k=5$, thus representing a situation in which different patients are often assigned to different clusters, making the assignment of patients to clusters unstable.

## Appendix 4. Stability of the RSC Results

The goal of this section is to investigate the stability of the clustering results with respect to the population size. Thus first the RSC clustering algorithm is applied to the two cohorts separately; in detail, the algorithm is applied to the 116 subjects from Padua and to the 56 subjects from St. Louis. Then the RSC algorithm is applied to the whole ischemic population, with the constraint of NIHSS>0, comprising 248 subjects.

Concerning the first case, the optimal number of clusters is 4 for Padua and 4 for St. Louis. The Sankey’s plot, displaying how patients clustered in Padua and St. Louis separately are mapped into the clusters obtained when the two cohorts are together, is shown in Fig. 15.

The Facial Palsy Cluster C3 (34 patients) obtained from PD+SL shared 24/25 patients of PD Cluster C4. The Motor Left Cluster C0 (27 patients) obtained from PD+SL has 9/9 patients from SL Cluster C3 and 15/16 patients from PD Cluster C0. The Motor Right Cluster C2 (26 patients) obtained from PD+SL has 10/11 patients from SL Cluster C1 and 16/24 patients from PD Cluster C2. The Language Cluster C4 (45 patients) obtained from PD+SL is mainly constituted of the 27/27 patients from PD Cluster C1 and other patients from SL Cluster C2 and 7/24 patients from PD Cluster C3. Finally, the heterogeneous cluster C1 obtained from PD+SL has 14/15 patients from SL Cluster C0 and 15/24 patients from PD Cluster C3 and the remaining from SL Cluster C2. In summary, patients found in the clusters when the cohorts were kept separate are found again in the clusters obtained with the combined cohorts.

Concerning instead the ischemic population, with the constraint of NIHSS$>0$, comprising 248 subjects, the optimal number of clusters found is 4, which radar charts are reported in Fig. 16.

The results here presented show high agreement, with the results presented in Fig. 4. In detail, Cluster 3, Cluster 1, and Cluster 0 here obtained, show NIHSS symptoms profile very similar to those shown in Fig. 4. The only difference here is that the language cluster is included in Cluster 2. This is reasonable for several reasons. First, here the optimal number of clusters is $k=4$, which is less respect the one found in the main manuscript $k=5$, so if all the other three clusters are the same, of course, two clusters have to be merged together. Secondly, the language cluster is the only one that can be adsorbed within another cluster, specifically in the Motor Right Cluster C2, since lesions for these two domains are lateralized on the same side of the brain. Finally, the other sub-optimal number of clusters is $k=7$; since there are many more clusters available, the language cluster (as shown in Cluster 4 Fig. 4) is retrieved again.

## Appendix 5. Voxel-wise Results

The statistical significant lesions shown in Fig. 5, are here visualized in Fig. 17 at different slices.

## Appendix 6. Distances

Five different distances have been used to calculate the distributions of the pair-wise similarities, together with GDM. These five distances are the most used in clustering as discussed in [55]. Since Manhattan and Euclidean distances are not normalized in the 0–1 range, the pair-wise distance has been normalized with respect to the maximum distance found in the dataset. Results are reported in Fig. 18.

## Code Availability

Code is available at https://github.com/MedMaxLab/nihss_clustering
